# The ethical, moral, and pragmatic rationale for brain augmentation

**DOI:** 10.3389/fnsys.2014.00130

**Published:** 2014-07-22

**Authors:** Vincent P. Clark

**Affiliations:** ^1^Psychology Clinical Neuroscience Center, University of New MexicoAlbuquerque, NM, USA; ^2^Department of Psychology, University of New MexicoAlbuquerque, NM, USA; ^3^Department of Neuroscience, University of New MexicoAlbuquerque, NM, USA; ^4^Mind Research Network and LBERIAlbuquerque, NM, USA

**Keywords:** TMS/tDCS, cognition, technology, evolution, brain

This opinion paper discusses the importance of augmentation in general and brain augmentation in particular, and considers the merits of some arguments that have been made against it. Augmentations are technologies created in order to improve upon human capabilities and characteristics. Such technologies have had a profound effect on our species, and their omnipresence might be said to distinguish us apart from every other species more-so than anything aside from our DNA. Augmentations are so essential to us, so widespread, that we often forget that they are artificial, and not truly a part of us. Indeed, if all augmentations were lost to us and could not be replaced, without any viable means to feed and protect ourselves, most humans would be dead in very a short time.

The need for augmentation is related in part to our poor biological adaptations for survival. We are born without claws, fur, and other characteristics that most animals require. What evolution has endowed us with instead is a large and complex nervous system. Our brains are metabolically expensive, requiring over 20% of our metabolism, while representing only 2% of our body mass (Kety, 1957; Sokoloff, 1960; Rolfe and Brown, 1997). Large brains are also difficult to grow, causing potential problems in childbirth as the baby's head must pass through the birth canal, and a long childhood is required to learn the minimum needed for survival and to become a member of society. However, they provide us with the means to compensate for our physical limitations. We can imagine, create, and manufacture a variety of augmentations. Housing and clothing for protection, agriculture for food, transportation, medicine, and many others. Such physical augmentations have provided us with a means to expand our livable environment to include most of the planet, including underwater and outer space, and they satisfy our biological and emotional needs far beyond what would otherwise be possible.

Augmentative technologies include not only physical but also cognitive enhancements. Similar to physical enhancements, cognitive enhancements provide us with capabilities beyond that provided by our nervous systems, to maintain or expand our perceptual, cognitive, and affective capabilities. This includes such devices as telescopes, microscopes, hearing aids, television, the internet, and myriad other devices that expand our senses and allow us to perceive much more than our immediate environment. Cognitive enhancements also include writing and mathematics, which have provided tremendous benefits, matched only by the potential of the computer age, which has transformed our world in just a few decades, and is likely to continue to do so.

Learning how to use any augmentation leads to adaptive changes in the nervous system as new memories are encoded and procedures are automated. By contrast, neuroenhancement changes the nervous system directly, augmenting cognition, and behavior from the inside out. Current forms of neuroenhancement include psychoactive substances, biofeedback, cognitive training, methods that promote physical health such as nutrition and exercise, mindfulness, electromagnetic methods of neurostimulation such as deep brain stimulation (DBS), transcranial magnetic stimulation (TMS) and transcranial direct current stimulation (tDCS) and others reviewed in Clark and Parasuraman (2014), and methods using ultrasound or near-infrared light. Indeed, neuroenhancement is not just “one thing” any more then exercise or learning is one thing, but instead covers a large and expanding array of different technologies.

Recent work in neuroenhancement has produced some surprising results. Work in our laboratory has shown that tDCS, a relatively simple technique that uses a 9-volt battery to produce a 1–2 mA current, led to large improvements in learning of a difficult visual perceptual task. Performance and d' (a measure of signal discriminability) were found to double while the false alarm rate was halved (Clark et al., 2012; Coffman et al., 2012b). This showed a dose-response effect (Clark et al., 2012), lasted for over 24 h (Falcone et al., 2012), and was not related to stimulation artifact or the type of experimental blinding used (Coffman et al., 2012b). TDCS also alters neurochemistry (Clark et al., 2011) and increases some forms of attention (Coffman et al., 2012a), which together and along with other effects may result in these large improvements in behavior. Other laboratories have found that tDCS can be used to increase working memory, explicit and implicit memory, perception and attention (reviewed in Coffman et al., 2014), with an average increase in effect size of about 0.9 between active and sham across studies. Using different methods of stimulation, these forms of cognition can also be reduced.

It should be clear to the reader by now that augmentation is essential to human existence, that physical-, cognitive-, and neuro-enhancement are all important to us, and that continued development of neuroenhancement technologies may lead to even more useful forms of brain augmentation. While there is some debate about its ultimate utility (Walsh, 2013), most evidence suggests that with further development, these many forms of neuroenhancement will likely lead to at least a few useful applications, and perhaps many. However, recent advances in neuroenhancement have also led to claims that some types are too unusual, immoral or unethical and should be banned or heavily managed. These arguments take a number of different forms. I will next discuss a subset of these.

(1) Cognitive augmentations may provide benefits only to those few who can afford them, thus further widening the social and cultural gap created by income differences (discussed in Hyman, 2011).

Different technologies for cognitive augmentation vary greatly in their relative cost. DBS can indeed be very costly, requiring neurosurgery to implant a carefully manufactured devise. However, other methods such as tDCS are relatively inexpensive, using technology that is already widely available for other purposes, electrodes made of inexpensive materials, and a single 9-volt battery that can operate the device for hours. So, while some methods of neuroenhancement may be too expensive for many people, other methods exist that are more affordable. I suggest here that simpler, cheaper and safer methods of neuroenhancement, and indeed all medical technologies, should be developed and attempted for treatment first, and only when these are proven to be inadequate should more expensive and potentially dangerous methods be attempted. This would lead to the most cost effective, safe, and beneficial treatments becoming more widely available, while reducing the potential harm of treatment side-effects.

(2) Cognitive augmentation might lead to an enhancement “arms race,” where all people are required to use enhancement in order to stay competitive (summarized in Hyman, 2011).

This concern may have some basis in fact. If we look at other forms of augmentation, those that are most useful are often adapted by the majority of people, to the detriment of those few who do not or cannot. Literacy provides an excellent example of this. Historically, literacy was limited to just a few, helping to maintain the sociopolitical structure, with those on top using literacy to maintain control. Once literacy was more widely disseminated, it helped to promote democracy and social change. Today, those who are illiterate are often relegated to lower-paying jobs, welfare, or crime to survive. Indeed, since literacy is so beneficial, it is most often caused by poor circumstances, rather than a personal choice. Might it be argued the literacy is unfair because it relegates most involuntary illiterates to the lowest levels of society, and therefore should be denied to all? If a form of brain-augmentation turned out to be as beneficial as literacy, would it be equally unreasonable to deny it to all in order to respect the choices of a few? I argue here that, as with literacy, mathematics, computer proficiency, and many other examples, an entire society should not be put at a disadvantage to appease those few who cannot or choose not to use an augmentation. Should there be additional costs in terms of safety or other issues, this becomes more complicated, and needs to be dealt with by weighing factual evidence carefully in terms of costs vs. benefits, and our commonly accepted sense of ethics. In order to do this adequately, published studies of neuroenhancement must use carefully controlled and replicable methods, provide statistics, and openly describeside effects and failures so that both costs and benefits can be well understood.

(3) Enhancements in one cognitive domain may lead to reductions in other domains (Brem et al., 2014).

This argument assumes that emphasizing one form of cognition must necessarily reduce others to maintain the total amount of cognitive function. While there is some evidence that decrements in one form of cognition can lead to enhancements in others (Luber and Lisanby, 2014), this hypothesis leads to a number of questions. For instance, is enhancement necessarily an increase in a specific cognitive “volume,” or instead might other mechanisms be involved, such as reducing neural “noise,” leading to increased neural SNR but without adding to cognitive load? Also, is total cognition stagnant, or does it vary over time? There is substantial evidence that it does, such as from birth to adulthood, and depending on state factors such as alertness, amount of sleep, nutrition, exercise, training and so on, and so enhancements that affect these may increase cognitive “volume” without reductions in other areas. Finally, if reductions are indeed found in other cognitive domains, the question arises of how important those domains are relative to those that are enhanced. In all cases, it's important to obtain reliable empirical data, and perform cost-benefit analyses before making firm decisions on whether cognitive- and brain-augmentations should be used.

(4) Uncertainty regarding safety is also an issue.

Potential dangers are present for all forms of brain augmentation, but the questions that should be asked are, “Is it as safe or safer than other methods currently in use?” and “Do the benefits outweigh the costs?” For some forms of neuroenhancement, their use might possibly cause harm. As an example, DBS produces damage during implantation, and can lead to bleeding, infection, and other issues that have resulted in the deaths of some patients (Rocha et al., 2014). However, most methods of neuroenhancement are unlikely to cause physical harm if used correctly. The use of any medical augmentation should be based on published clinical trials when available, using methods approved by IRBs or other governing bodies. There are some important differences between pharmaceuticals and tDCS that suggest tDCS should be safer. TDCS can be ended in a few seconds, compared with a “wash out” period of days or weeks for some pharmaceuticals. TDCS can be directed to specific points on the head, while avoiding other organs such as liver, heart and kidneys, where deleterious side effects of pharmaceuticals can sometimes occur. Also the chance of forming unexpected chemical side products from interactions with other concurrent treatments is reduced. Overall, tDCS has far fewer and less dangerous side effects, limited to redness/irritation under the electrodes, mild tingling, itching, heat and less commonly fatigue, transient headaches, nausea, and insomnia (Poreisz et al., 2007). Furthermore, brain stimulation for pain treatment is much less likely to lead to addiction when compared with opiates. Based on these qualities, tDCS appears safer than pharmaceuticals for most applications. Once effective methods of brain stimulation for clinical treatment are developed in smaller studies, large scale clinical trials need to be performed in order to better understand their costs and benefits relative to the current standard of care. As an example, TDCS shows great potential for the treatment of a variety of illnesses, but few large-scale clinical trials have been performed thus far.

(5) A broader argument involves the potential risks imposed by our increasing dependence on modern technologies that might have unforeseen negative effects, resulting in our ultimate demise (Rees, 2003).

While the combined side effects of our technological world, such as pollution, population expansion, diminishing resources, and continued warfare may end our domination of Earth as soon as it began, at present we are at our peak in many ways. The possibility that an otherwise safe augmentative technology might lead to our demise is impossible to predict. We cannot see into the future, but we can look for patterns that may help us to estimate the likelihood of future events. Some technologies, such as landmines and nuclear weapons, can be argued to produce much more harm than good and should be banned. However, nearly anything can be used with the intention of doing harm. From sharpened rocks to rockets, from ancient writing to modern computers, even the wheel, nearly all technologies have been used to support warfare or to cause harm in some way. By contrast, cognitive- and brain-augmentation technologies have provided many benefits to us thus far, and will likely continue to do so. Our success has a species has primarily been derived through our intelligence and cognitive capabilities. Enhancing these further through the benefits of cognitive- and brain-augmentation, we might one day develop better, safer and more sustainable technologies to reduce pollution, better manage resources, reduce the need for or find alternatives to warfare, and improve our chances of survival overall, rather than reducing them.

## Summary

We can say with certainty that our species would be unlikely to exist as it does today without augmentation. Cognitive- and neuroenhancements are just the latest in a long line of augmentations that have helped our species to survive and flourish. While there are some valid concerns, the benefits of brain augmentations like tDCS will likely far outweigh their costs when properly used. The continued pursuit of cognitive- and neuro-enhancement will help us to improve our quality of life, reduce suffering from brain and mental illness, and enhance our chances of survival long-term.

### Conflict of interest statement

The author declares that the research was conducted in the absence of any commercial or financial relationships that could be construed as a potential conflict of interest.

## References

[B1a] Brem A. K. Fried P. J. Horvath J. C. Robertson E. M. Pascual-Leone A. (2014). Is neuroenhancement by noninvasive brain stimulation a net zero-sum proposition? Neuroimage 85 Pt 3, 1058–1068 10.1016/j.neuroimage.2013.07.038 23880500 PMC4392930

[B1] Clark V. P. Coffman B. A. Mayer A. R. Weisend M. P. Lane T. D. R. Calhoun V. D. (2012). TDCS guided using fMRI significantly accelerates learning to identify concealed objects. Neuroimage 59, 117–128 10.1016/j.neuroimage.2010.11.036 21094258 PMC3387543

[B2] Clark V. P. Coffman B. A. Trumbo M. C. Gasparovic C. (2011). Transcranial direct current stimulation (tDCS) produces localized and specific alterations in neurochemistry: a 1H magnetic resonance spectroscopy study. Neurosci. Lett. 500, 67–71 10.1016/j.neulet.2011.05.244 21683766

[B3] Clark V. P. Parasuraman R. (2014). Neuroenhancement: enhancing brain and mind in health and in disease. Neuroimage 85, 889–894 10.1016/j.neuroimage.2013.08.071 24036352

[B4] Coffman B. A. Clark V. P. Parasuraman R. (2014). Battery powered thought: a review of methods for cognitive enhancement using transcranial direct current stimulation. Neuroimage 85, 895–908 10.1016/j.neuroimage.2013.07.083 23933040

[B5] Coffman B. A. Trumbo M. C. Clark V. P. (2012a). Enhancement of object detection with transcranial direct current stimulation is associated with increased attention. BMC Neurosci. 13:108 10.1186/1471-2202-13-108 22963503 PMC3494452

[B6] Coffman B. A. Trumbo M. C. Flores R. A. Garcia C. M. van der Merwe A. J. Wassermann E. M. (2012b). Impact of tDCS on performance and learning of target detection: interaction with stimulus characteristics and experimental design. Neuropsychologia 50, 1594–1602 10.1016/j.neuropsychologia.2012.03.012 22450198

[B7] Falcone B. Coffman B. A. Clark V. P. Parasuraman R. (2012). Transcranial direct current stimulation augments perceptual sensitivity and 24-hour retention in a complex threat detection task. PLoS ONE 7:e34993 10.1371/journal.pone.0034993 22511978 PMC3325218

[B8] Hyman S. E. (2011). Cognitive enhancement: promises and perils. Neuron 69, 595–598 10.1016/j.neuron.2011.02.012 21338872

[B9] Kety S. S. (1957). The general metabolism of the brain *in vivo*, in Metabolism of The Nervous System, ed Richter D. (London: Pergamon), 221–237

[B9a] Luber B. Lisanby S. H. (2014). Enhancement of human cognitive performance using transcranial magnetic stimulation (TMS). Neuroimage 85 Pt 3, 961–970 10.1016/j.neuroimage.2013.06.007 23770409 PMC4083569

[B10] Poreisz C. Boros K. Antal A. Paulus W. (2007). Safety aspects of transcranial direct current stimulation concerning healthy subjects and patients. Brain Res. Bull. 72, 208–214 10.1016/j.brainresbull.2007.01.004 17452283

[B11] Rees M. (2003). Our Final Century. London: Heineman

[B12] Rocha S. Monteiro A. Linhares P. Chamadoira C. Basto M. A. Reis C. (2014). Long-term mortality analysis in Parkinson's disease treated with deep brain stimulation. Parkinson's Dis. 2014:717041 Available online at: http://www.hindawi.com/journals/pd/2014/717041/ 10.1155/2014/717041 24772365 PMC3960527

[B13] Rolfe D. F. S. Brown G. C. (1997). Cellular energy utilization and molecular origin of standard metabolic rate in mammals. Physiol. Rev. 77, 731–758 9234964 10.1152/physrev.1997.77.3.731

[B14] Sokoloff L. (1960). The metabolism of the central nervous system *in vivo*, in Handbook of Physiology, Section I, Neurophysiology, Vol. 3, eds Field J. Magoun H. W. Hall V. E. (Washington, DC: American Physiological Society), 1843-1864

[B15] Walsh V. Q. (2013). Ethics and social risks in brain stimulation. Brain Stimul. 6, 715–717 10.1016/j.brs.2013.08.001 24021548

